# Molecular Evidence for Precursors of Sjögren’s Foci in Histologically Normal Lacrimal Glands

**DOI:** 10.3390/ijms20010223

**Published:** 2019-01-08

**Authors:** Austin K. Mircheff, Yanru Wang, Billy X. Pan, Leili Parsa, Prachi Nandoskar, Chuanqing Ding

**Affiliations:** 1 Department of Physiology & Neuroscience, Keck School of Medicine, University of Southern California, Los Angeles, CA 90033, USA; yanruwan@gmail.com; 2 Department of Ophthalmology, Keck School of Medicine, University of Southern California, Los Angeles, CA 90033, USA; billyxiapan@gmail.com; 3 Department of Cell & Neurobiology, Keck School of Medicine, University of Southern California, Los Angeles, CA 90033, USA; p_leily@yahoo.com (L.P.); prachi.nandoskar@gmail.com (P.N.); cding@usc.edu (C.D.)

**Keywords:** autoimmunity, lacrimal gland, Sjögren’s syndrome

## Abstract

Understanding the formation of Sjogren’s lymphocytic infiltrates could permit earlier diagnosis and better outcomes. We submitted gene transcript abundances in histologically normal rabbit lacrimal glands to principal component analysis. The analysis identified a cluster of transcripts associated with Sjögren’s foci, including messenger RNAs (mRNAs) for C–X–C motif chemokine ligand 13 (CXCL13) and B-cell activating factor (BAFF), which dominated the major principal component. We interpreted the transcript cluster as the signature of a cluster of integrally functioning cells. Pregnancy and dryness increased the likelihood that the cluster would develop to high levels, but responses were subject to high levels of stochasticity. Analyzing microdissected samples from high- and low-cluster-level glands, we found that certain transcripts, including mRNAs for C–C motif chemokine ligand 21 (CCL21), CXCL13, cluster of differentiation 4 (CD4), CD28, CD25, BAFF, and interleukin 18 (IL-18) were significantly more abundant in immune cell clusters (ICs) from the high-cluster-level gland; mRNAs for CCL2, CD25, and IL-1RA were significantly more abundant in acinus-duct axis samples; mRNAs for CCL4, BAFF, IL-6, and IL-10 were more abundant in some acinus-duct samples; cells with high prolactin immunoreactivity were more frequent in interacinar spaces. In conclusion, integrated functional networks comprising Sjögren’s infiltrates, such as ICs, acinar cells, ductal cells, and interacinar cells, can form in histologically normal glands, and it is feasible to detect their molecular signatures.

## 1. Introduction

Sjögren’s syndrome is an autoimmune epithelitis characterized by production of antibodies and focal lymphocytic infiltrates that cause severe dysfunction of the lacrimal glands and salivary glands. The secretory dysfunction leads to ocular surface and oral pathology and symptoms. When manifestations of the autoimmune processes arise in extraglandular sites, such as the kidneys, vasa vasorum, and peripheral vessels, they further impair quality of life and significantly increase risks of life-threatening vasculitides and non-Hodgkin’s mucosa-associated lymphoid tissue (MALT) lymphomas.

An understanding of how the disease processes develop and evolve could improve diagnosis and treatment. However, the etiopathogenesis of Sjögren’s syndrome remains a formidable challenge. Sjögren’s syndrome comprises two major subtypes, i.e., primary Sjögren’s syndrome and secondary Sjögren’s syndrome [1], and both subtypes present in multiple clinical phenotypes. The cellular and molecular processes that underlie the clinical subtypes are diverse. Primary Sjögren’s infiltrates may be either T-cell predominant or B-cell predominant [2], and may have either positive- or negative type I interferon (IFN) signatures [3]. B-cell predominant infiltrates may have or lack germinal centers [4]. These features imply that Sjögren’s syndrome may have multiple etiologies and may develop along multiple pathogenic pathways.

Identified risk factors offer clues to Sjögren’s syndrome etiology. It is known for some time that certain human leukocyte antigen (HLA) class II molecule variants favor B-cell responses to the anti-Sjögren’s-syndrome-related A (Ro/SSA) and anti-Sjögren’s-syndrome-related B (La/SSB) autoantigens, formation of lymphocytic infiltrates with germinal centers, and risks for MALT lymphomas and vasculitides, evidently because the high-risk HLA class II variants have high affinities for Ro/SSA and La/SSB epitopes [5,6,7]. Additional genetic polymorphisms reported to be associated with risk for Sjögren’s syndrome include variants of interleukin 10 (IL-10) [8,9,10], tumor necrosis factor (TNF) [11], antigen peptide transporter 2 (TAP2) [12], and 2′-5′-oligoadenylate synthetase 1 (OAS1) [13]. Female sex is a well-known risk factor [14], and parity was also reported to be a risk factor [15,16]. The concordance rate for monozygotic twins was estimated to be low [17]. Therefore, interactions between genetic predisposition, sex, and parity are not sufficient to determine Sjögren’s etiology. Other phenomena, assumed to be environmental in nature, must be involved. 

In 1983, after observing that thyrocytes in Graves’ disease patients express HLA class II molecules [18], Bottazzo, Pujol-Borrell, and colleagues proposed that induction of aberrant HLA class II molecule expression allows non-immune system cells to present autoantigen epitopes directly to T cells and initiate formation of autoimmune lesions [19]. In the time since, epithelial cells in labial salivary glands of patients with Sjögren’s syndrome were confirmed to express HLA class II molecules [20,21,22], as well as HLA class I molecules. [23]. They were also shown to express cluster of differentiation 80 (CD80) and CD86 [24], which provide the costimulatory signals necessary for T cells to become activated after antigenic stimulation; the costimulatory molecule CD40 [25]; several chemokines (C–C motif chemokine ligand 3 (CCL3), CCL4, CCL5, CCL17, CCL21, CCL22, CCL28, C–X–C motif chemokine ligand 8 (CXCL8), CXCL9, CXCL10, CXCL12, CXCL13, and CX3CL1) [26,27,28,29,30,31,32,33]; several cell adhesion molecules (CD2, intercellular adhesion molecule 1 (ICAM-1), lymphocyte function-associated 1 (LFA-1), and LFA-3) [34]; and several cytokines (B-cell activating factor (BAFF), granulocyte-macrophage colony-stimulating factor (GM-CSF), IFN-γ, IL-lα, IL-1β, IL-2, IL-6, IL-10, IL-18, IL-28/IL-29, transforming growth factor beta (TGF-β), and TNF-α) [34,35,36,37,38,39]. 

Many of the immune response-related molecules expressed by epithelial cells of salivary glands with Sjögren’s lesions also are expressed by epithelial cells in salivary glands from healthy control subjects, but generally at much lower levels than in affected glands. Therefore, the data suggest that exposures to certain risk factors increase the probability that exocrine gland epithelial cells will upregulate their expression of genes that cause them to either (1) function as surrogate antigen presenting cells, or (2) create local microenvironments where interactions between professional antigen-presenting cells, autoreactive T cells, and autoreactive B cells are likely lead to immune cell activation and self-organization of ectopic lymphoid structures. 

Working with histologically normal lacrimal glands from healthy rabbits, we confirmed Frey and coworkers’ finding that lacrimal gland epithelial cells express exhibit immunohistochemical positivity for prolactin (PRL) [40], which has pleiotropic actions as a cytokine [41,42,43,44]. We found that corneal injury, corneal adenovirus infection, and pregnancy altered levels of PRL immunoreactivity [45,46]. We also found that lacrimal glands express messenger RNA (mRNA) for PRL and also mRNAs for class II major histocompatibility complex (MHC II), CD80, and CD86, numerous cytokines, and numerous chemokines. The abundances of many transcripts exhibited considerable gland-to-gland variability. Some of the abundance variations appeared to be related to the dryness, while others were related to the temperatures and to the environment the animals experienced prior to study [47,48]; additional variability was induced by the hormonal environment of pregnancy [49]. However, much of the variability appeared related to stochastic phenomena localized within glands. Pearson’s analysis of the variability showed that variations of clusters of transcripts were significantly correlated. Principal component analyses confirmed the major correlation cluster and indicated that certain transcripts might be expressed by two or more correlation clusters in the same gland. We interpreted the transcript correlation clusters as signatures of clusters of cells that were interacting with each other coordinately.

The major correlation cluster was of interest, as it included mRNA for BAFF, a B-cell mitogen and activating factor, and mRNAs for the B-cell chemokine, CXCL13, and the T-cell cytokine, CCL21, all associated with Sjögren’s lymphocytic foci. A preliminary laser capture microdissection of glands from a nulliparous animal confirmed that epithelial cells expressed a number of immune response-related gene transcripts, that immune cells in small clusters expressed certain other transcripts, and that both epithelial cells and immune cells expressed certain transcripts. Notably, the immune cell clusters, or “accumulations”, were too small to be identified as Sjögren’s foci. These characteristics suggest that epithelial cells and immune cells can engage in multiple signaling interactions with each other, forming localized networks that might expand over time to eventually manifest as histopathologically identifiable Sjögren’s lesions.

We collated and analyzed data from our previously published studies to discern how age, pregnancy, and exposures varying ambient conditions influence the formation of correlation clusters. We also analyzed the abundances of selected transcripts in samples of acinar cells, intralobular duct cells, interlobular duct cells, intralobar duct cells, and clustered immune cells obtained by laser capture microdissection of three glands from term-pregnant animals. Two glands were representative of the subgroup of glands in which the Sjögren’s-resembling transcript correlation cluster was present at low levels, and one gland was representative of the subgroup in which the Sjögren’s-resembling transcript correlation cluster was present at high levels. 

We found that epithelial cells rarely express mRNA for MHC II, but frequently express mRNA for CD1d, which presents both bacterial and autologous glycolipids invariant α-chain natural killer (NK) T cells. They even more frequently express mRNA for MHC I, which presents epitopes of intracellular proteins, both viral and autologous, to CD8^+^ T cells. We also found that samples of the epithelial segments heterogeneously express costimulatory molecules, chemokines, and cytokines. These findings support the hypothesis that epithelial cells in histologically normal glands can interact with clusters of immune cells to comprise networks with stochastically varying cellular compositions and transcript expression profiles. Environmental and hormonal exposures increase the likelihoods that small networks will expand and, perhaps, evolve as ectopic lymphoid structures of different phenotypes. The ability to detect molecular signatures of the small networks in histologically normal glands may have important implications for the future diagnosis and proactive treatment of incipient Sjögren’s syndrome.

## 2. Results

### 2.1. Systemic and Stochastic Variations in Immune Response-Related Gene Transcript Expression

**Figure 1:**
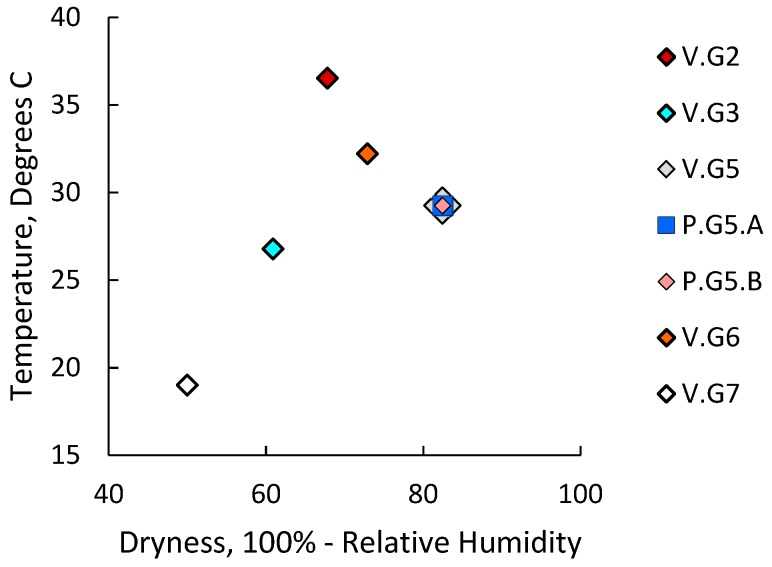
Experimental group exposures to ambient dryness and high temperature. Values presented for groups V.G2, V.G3, V.G5, P.G5, and V.G6 are average daily high temperatures and average daily maximum degrees of dryness (100%—relative humidity) the groups experienced in the barrier-free facility where they were housed during the 30 days before transport to University of Southern California (USC) Vivaria. Values for group V.G7 are average temperatures and degrees of dryness the group experienced in the controlled environments at Covance Research Products Vivaria and USC Health Sciences Campus (HSC) Vivaria. Groups V.G2, V.G3, V.G5, and V.G6 were nulliparous and 20 weeks old. Group V.G7 was nulliparous and 52 weeks old. Group P.G5 was 29 days pregnant and 20 weeks old.

To compare responses to exposures to varying combinations of ambient dryness and temperature, the hormonal environment of pregnancy, and the transition from young adulthood to mature adulthood, we collated abundance data for transcripts that were measured in all three previous studies and submitted the data to principal component analysis (PCA). Groups V.G2, V.G3, V.G5, P.G5, and V.G6 were 20 weeks old and were raised and housed in a barrier-free facility, where they experienced different environmental exposures. Group V.G7 was 52 weeks old and was raised and housed in barrier facilities. Group P.G5 was term-pregnant; the other groups were nulliparous. Figure 1 presents the average daily high temperatures and average maximum degree of dryness that the six groups experienced. 

**Figure 2:**
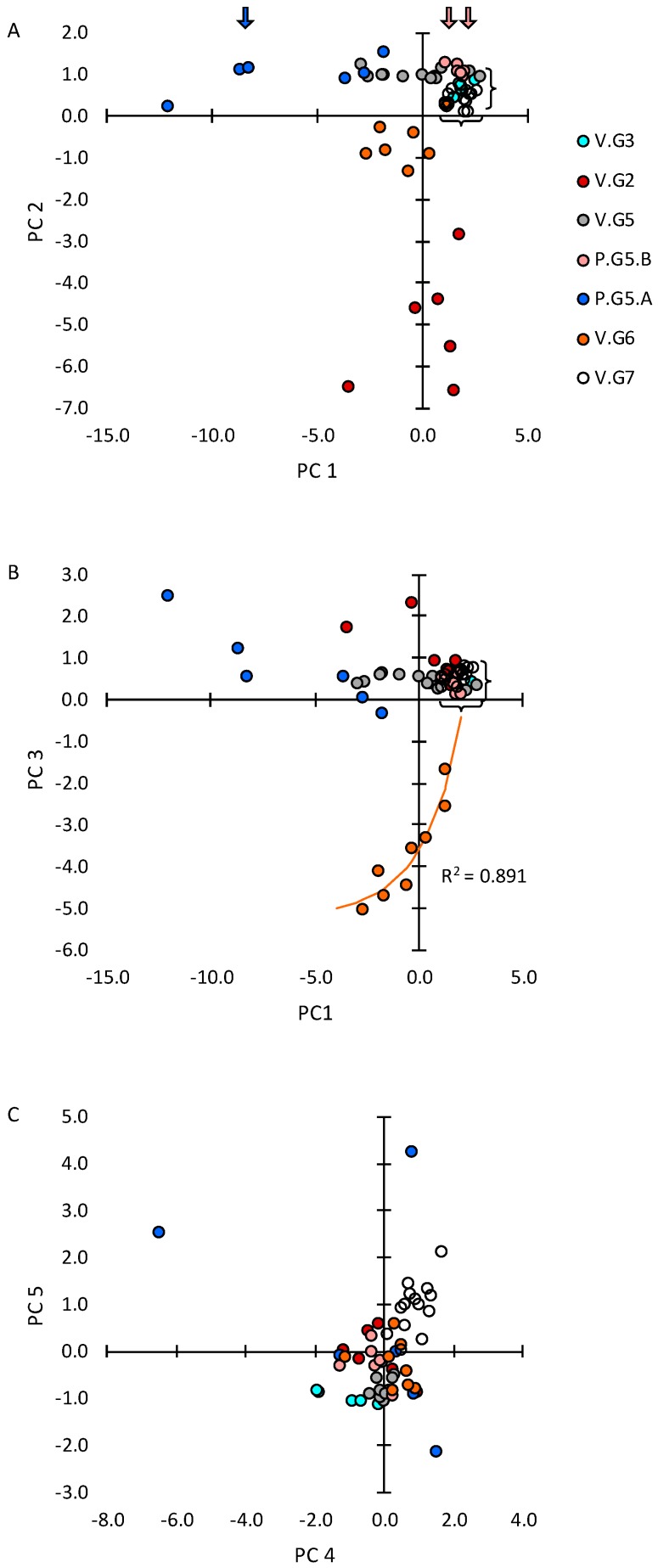
Principal component (PC) projections of individual glands from all groups determined by principal component analysis of the collated abundances of 20 transcripts that were assayed in all whole-gland samples. (**A**) PC2 v PC1; (**B**) P3 v PC1; (**C**) PC5 v PC4. A total of 72 transcripts were assayed in whole-gland samples from groups V.G5 and P.G5 [49]. Discrepancies between principal component values from that dataset and the collated dataset resulted primarily from the data from groups V.G2 and V.G6. Arrows indicate PC1 projections of glands that were selected for microdissection.

The individual glands’ projections with respect to the significant principal components are presented in Figure 2. The glands from the two groups that experienced the most benign ambient conditions (i.e., V.G7 and V.G3) clustered closely together within narrow ranges of PC1, PC2, and PC3 projections, indicated by brackets in Figure 2A,B. We interpreted these as empirical normal ranges. The glands from groups V.G2, V.G5, and V.G6 (i.e., nulliparous groups that experienced hotter, drier, or hotter and dryer conditions) were distributed more dispersedly with respect to PC1. Projections of some V.G2 and V.G5 glands fell within the empirical normal range; projections of other V.G2 and V.G5 glands, and of all V.G6 glands, fell well below the empirical normal range. The glands from group P.G5 (i.e., the term-pregnant animals) exhibited the most dispersed distributions with respect to PC1; one clearly-defined subgroup of glands exhibited positive PC1 projections within the empirical normal range; the other subgroup exhibited negative PC1 projections that extended well beyond the ranges of the glands from any of the other groups. We designated the respective subgroups P.G5.B and P.G5.A, and we selected one gland from each subgroup for further analysis.

The V.G3, V.G5, V.G6, and P.G5.A glands clustered together with respect to their PC4 and PC5 projections, again defining empirical normal ranges (Figure 2C). The V.G7 glands fell outside the empirical normal ranges, possibly reflecting an influence of the animals’ transition from young adulthood to maturity. Four of the P.G5 glands fell outside or well outside the empirical normal ranges, one in each quadrant, possibly reflecting stochastic responses to the hormonal environment of pregnancy.

**Figure 3:**
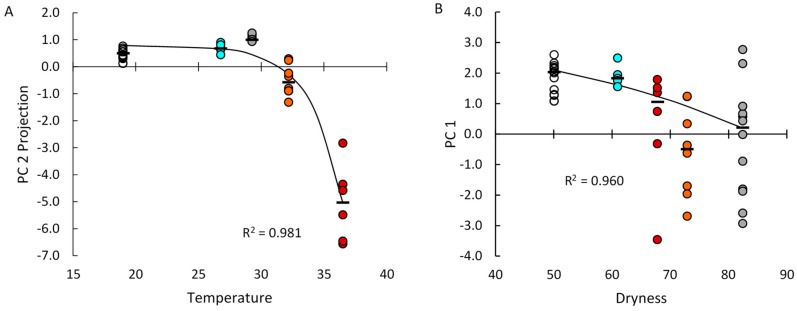
Relationships between median principal component projections, temperature, and dryness for glands from the nulliparous animals. (**A**) PC2 projections v mean daily high temperature; (**B**) PC1 PC 1 projections v mean daily high degree of dryness. (**−**, median projections; other symbols as in Figure 3A).

Median abundances of numerous transcripts in the glands from groups V.G2, V.G3, V.G5, and V.G6 could be described by heuristics related to the average daily high temperature or the average daily maximum degree of dryness the animals experienced [47,48]. Therefore, we analyzed the relationships between the environmental variables and the principal component projections of the glands from all five groups of nulliparous animals described in this study. As shown in Figure 3A, the median PC2 projections could be modeled as decreasing exponentially with exposure to increasing daily high temperatures (*R^2^* = 0.981). As shown in Figure 3B, the median PC1 projections could be modeled as decreasing exponentially with exposure to increasing degrees of dryness (*R^2^* = 0.960). The median PC1 projection of group V.G6 glands was notably displaced from the exponential growth model prediction. The V.G6 glands showed a significant exponential relationship between decreasing PC1 projections and increasing PC3 projections (Figure 2B, *R^2^* = 0.891), suggesting that a phenomenon related to the PC3 projections displaced their PC1 projections above the value predicted in Figure 3B.

**Figure 4:**
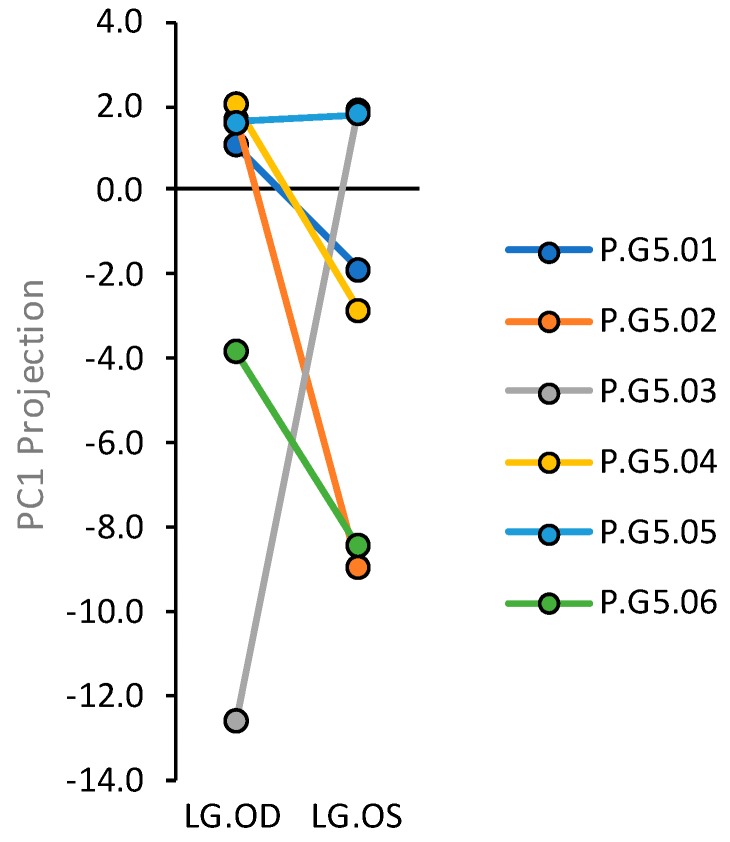
PC1 projections of right eye-associated (oculus dextrus (OD)) and left eye-associated (oculus sinister (OS)) glands from group P.G5.

The abundances of numerous transcripts exhibited low concordances between right eye (oculus dextrus (OD))-associated and left eye (oculus sinister (OS))-associated lacrimal glands from the group V.G5 animals [47]. To assess the relative contributions that systemic factors and strictly local stochastic factors made to PC1 projections of the P.G5.B and P.G5.A glands, we plotted PC1 projections of the companion right eye OD-associated and left eye OS-associated glands from each group P.G5 animal (Figure 4). Glands P.G5.05.OD and P.G5.05.OS were the only companions that exhibited similar PC1 projections. The poor concordances between companion glands indicate that strictly local stochastic factors contribute substantially to PC1 projection variations.

Table 1 presents the transcript loadings identified by principal analysis of the collated data. Messenger RNAs for IL-1α, IL-1β, IL-6, IL-10, CCL2, CCL4, CCR5, CXCL13, CD4, CD8, CD28, cytotoxic T-lymphocyte-associated antigen 4 (CTLA-4), BAFF, MHC II, and PRL contributed negative loadings to PC1 in this analysis. Many of these transcripts correlated strongly with each other when we submitted the complete datasets for the V.G2, V.G3, V.G5, and V.G6 glands to separate Pearson’s tests [47]. They also tended to contribute strong negative loadings to the first principal component when we submitted the complete datasets for the V.G7 glands [50] and the V.G5 and P.G5 glands to separate to principal component analyses [49]. Exceptions to this generalization resulted from having collated data from groups V.G2 and V.G6 together with data from the other groups.

### 2.2. Distribution of Transcript Expression among Epithelial Segments and Immune Cells

We used laser capture methodology to microdissect gland P.G5.06.OS, a representative subgroup P.G5A gland, and glands P.G5.01.OD and P.G5.03.OS, representative of subgroup P.G5.B. The selected glands are indicated by arrows in Figure 2A. We assayed abundances of selected transcripts in all samples from gland P.G5.06.OS and in immune cell clusters from gland P.G5.01.OD; we also assayed abundances of a subset of the selected transcripts in samples of acinar cells, intralobular duct cells, interlobular duct cells, and intralobar duct cells from gland P.G5.03.OS. The transcripts we assayed in the microdissected samples were a subset of the transcripts measured in whole-gland samples of the V.G5 glands, P.G5.A glands, and P.G5.B glands [49]. As this subset was larger than the subset analyzed in Figure 2 and Figure 3, we interpreted the findings with reference to the large sets of principal component projections and loadings identified by principal component analysis of the complete set of transcripts in samples of the V.G5 glands, P.G5.A glands, and P.G5.B glands [49], rather than the projections in Figure 2 and Figure 3 and the loadings in Table 1.

**Figure 5:**
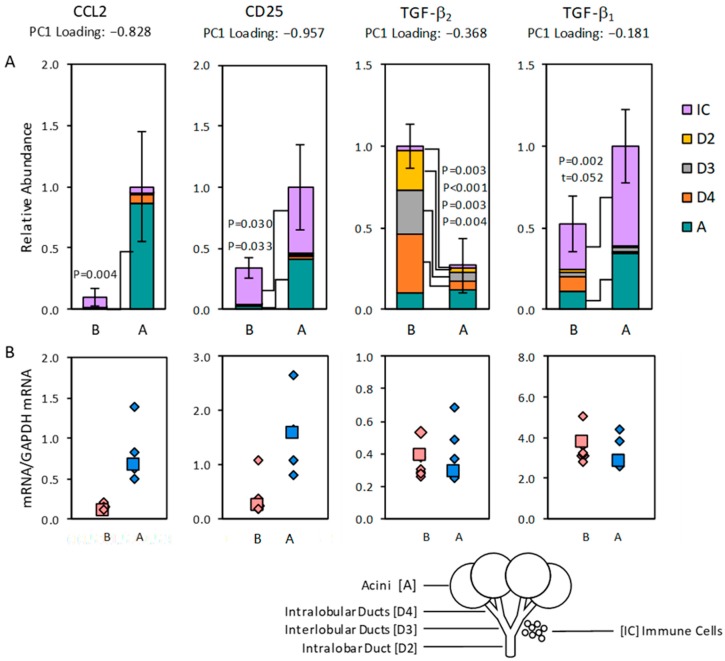
(**A**) Modeled abundances of selected transcripts in samples of immune cells (IC), intralobar duct cells (D2), interlobular duct cells (D3), intralobular duct cells (D4), and acinar cells (A) microdissected from representative subgroup P.G5.B glands (positive PC1 projection in Figure 2) and a representative subgroup P.G5.A gland (negative PC1 projection in Figure 2). Error bars indicate pooled standard errors for IC and the four epithelial elements. The transcripts selected showed statistically significant differences between epithelial segments from the P.G5.A gland and the P.G5.B glands (significant *p*-values and *p*-values indicating trends (t) are as indicated). (**B**) Measured abundances of the transcripts in whole-gland samples of the P.G5.B and P.G5.A subgroup glands. Glands that were microdissected are indicated by large-font symbols.

Many transcripts were present at detectable levels in acinar cell and duct cell samples, as well as in immune cell cluster samples. To estimate approximate relative amounts in acini, the duct segments, and immune cells, we used a model that weighed abundances in acinar cell, intralobular duct cell, interlobular duct cell, intralobar duct cell, and immune cell cluster samples by factors of 0.80, 0.04, 0.04, 0.04, and 0.08. The modeled amounts of mRNAs for CCL2 and CD25 were higher for gland P.G5.06.OS than the modeled amounts of P.G5.B glands (Figure 5). These relationships recapitulated the relationships between the transcripts’ abundances in the whole-gland samples. Therefore, the differences between abundances in the P.G5.A and P.G5.B whole-gland samples can be attributed to significantly higher abundance of mRNA for CCL2 in acinar cells from gland P.G5.06.OS, and to significantly higher abundances of mRNA for CD25 in both immune cells and acinar cells from gland P.G5.06.OS. These findings indicate that acinar cells contributed mRNA for CCL2, and both acinar cells and immune cells contributed mRNA for CD25 to the negative PC1-loading transcript cluster.

The modeled amount of mRNA for TGF-β_2_ was lower for gland P.G5.06.OS than for the P.G5.B glands, again recapitulating the relationship between the transcript’s abundances in the whole-gland samples. The difference can be attributed to significantly lower abundances in intralobular duct cells, interlobular duct cells, intralobar ducts cells, and immune cells from gland P.G5.06.OS. 

In contrast to the abundances of mRNAs for CCL2, CD25, and TGF-β_2_, the modeled abundances of mRNA for TGF-β_1_ in gland P.G5.06.OS were significantly higher than the modeled abundances in the P.G5.B glands, contrary to the relationships between the abundances measured in the whole-gland samples (Figure 5). It is possible that a cell type that we did not sample by laser capture microdissection of the P.G5.B gland expressed high levels of the mRNA for TGF-β_1_.

**Figure 6:**
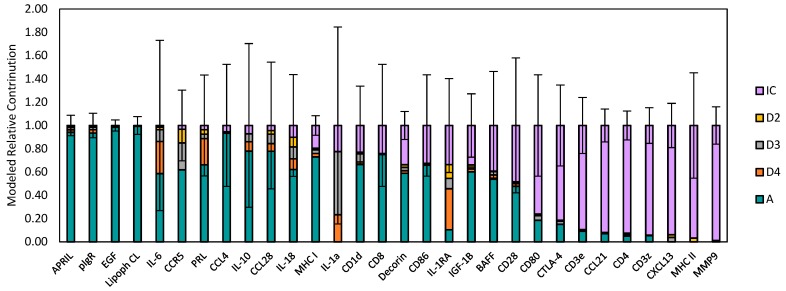
Modeled abundances of transcript assayed in microdissected samples from the P.G5.A gland (negative PC1 projection in Figure 2); values are normalized to total mean modeled abundances. Error bars indicate pooled normalized standard errors for IC and the four epithelial elements. Transcripts depicted in Figure 5 are not included.

Additional transcripts were also present at detectable levels in epithelial segment and immune cell samples (Figure 6). Messenger RNAs for a proliferation-inducing ligand (APRIL), polymeric immunoglobulin receptor (pIgR), epidermal growth factor (EGF), and lipophilin CL were predominantly expressed in acinar cells; none of these transcripts contributed to the PC1-negative loading transcript cluster [49]. Messenger RNAs for CD3ε, CCL21, CD4, CD3ζ, CXCL13, and MHC II were predominantly expressed in immune cell clusters, as discussed further below.

Other transcripts were expressed at appreciable levels in both epithelial segments and in immune cell clusters. In general, the modeled abundances of the transcripts that were expressed by both epithelial cells and immune cells exhibited much larger variances than the transcripts that were predominantly expressed by either acinar cells and or immune cells. Consequently, apparent differences between modeled abundances in the epithelial segment samples were often not statistically significant. Therefore, the findings indicate that acinar cells, intralobular duct cells, interlobular duct cells, and intralobar duct cells expressed detectible levels of the immune response-related gene transcripts, but they tended to be extremely heterogeneous with respect to the levels at which they expressed the transcripts. Messenger RNA for CCL4 appeared to be more abundant in acinar cells from gland P.G5.06.OS than in acinar cells from the P.G5.B gland. Similarly, mRNAs for BAFF, IL-6, and IL-10 appeared to be more abundant in both acinar cells and duct segment cells from gland P.G5.06.OS, but they exhibited large variances, and differences from the corresponding epithelial segments from the P.G5.B gland were not statistically significant. Therefore, it appears that some acini and duct segments contributed mRNAs for CCL4, BAFF, IL-6, and IL-10 to the PC1-negative loading transcript cluster. Acini and duct segments also expressed mRNAs for CCL28 and may have contributed it to the PC1-negative loading transcript cluster.

### 2.3. Transcript Expression in Immune Cell Clusters

**Figure 7:**
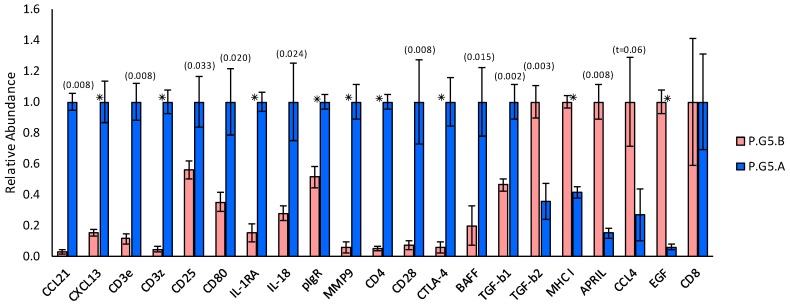
Relative transcript abundances in immune cell cluster samples microdissected from the subgroup P.G5.B gland (positive PC1 projection in Figure 2) and subgroup P.G5.A gland (negative PC1 projection in Figure 2). Only transcripts showing statistically significant differences are shown. Error bars indicate standard errors. * indicates *p* < 0.001; larger *p*- and *t*-values are given in parentheses. Values are normalized to the value; therefore, differences represent fold-changes. Mean abundances of messenger RNA (mRNA) for cluster of differentiation 8 (CD8) were similar in immune cell cluster samples from the two glands.

Immune cell cluster samples from gland P.G5.06.OS differed significantly from immune cell cluster samples from the P.G5.B gland with respect to the levels at which they expressed 20 of the assayed transcripts (Figure 7). Messenger RNAs for CCL21, CXCL13, CD3ε, CD3ζ, CD25, CD80, IL-1RA, IL-18, pIgR, matrix metalloproteinase 9 (MMP-9), CD4, CD28, CTLA-4, BAFF, and TGF-β_1_ were significantly more abundant in immune cell clusters from gland P.G5.06.OS. Messenger RNAs for TGF-β_2_, MHC I, APRIL, and EGF were significantly less abundant in immune cell clusters from gland P.G5.06.OS, and there was a trend (*p* = 0.06) toward a lower abundance of mRNA for CCL4 in immune cells from gland P.G5.06.OS. Notably, the transcripts that were less abundant in immune cell clusters from gland P.G5.06.OS were predominantly expressed by epithelial cells, rather than by immune cell clusters (Figure 5 and Figure 6).

Of the transcripts that were more highly expressed in immune cells from gland P.G5.06.OS, mRNAs for CCL21, CD4, CD25, CD25, CTLA-4, BAFF, and MMP-9 contributed strong negative loadings to PC1 [49]. These findings indicate that immune cell clusters contributed mRNAs for CCL21, CD4, CD25, CD25, CTLA-4, BAFF, and MMP-9 to the PC1-negative loading transcript cluster and, therefore, participated in a functional network with acinar cells contributing mRNA for CCL2, additional mRNA for CD25 and, presumably, mRNAs for CCL4, BAFF, IL-6, and IL-10.

**Figure 8:**
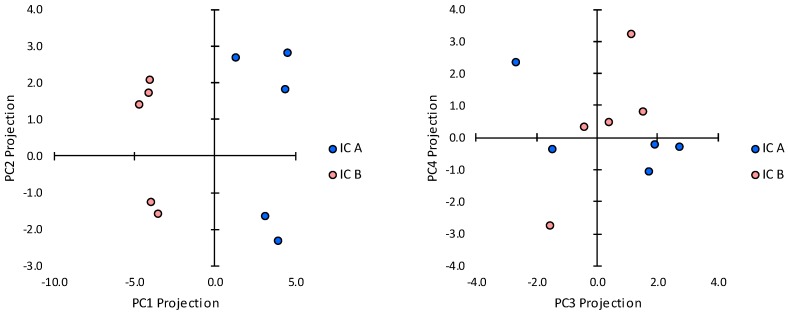
Principal component projections of immune cell cluster samples microdissected from (**A**) the subgroup P.G5.B gland (positive PC1 projection in Figure 2) and (**B**) the subgroup P.G5.A gland (negative PC1 projection in Figure 2).

The findings in Figure 5, Figure 6 and Figure 7 indicate that immune cell clusters in gland P.G5.06.OS contained an admixture of cells from other transcript clusters in addition to the PC1-negative loading cluster. Transcripts that contributed relatively weak negative loadings to PC1, i.e., mRNAs for CD3ζ, CD80, and CXCL13 [49], were also significantly more abundant in immune cell clusters in gland P.G5.06.OS. Notably, mRNA for CD3ζ contributed a strong positive loading, and mRNAs for CD80 and CXCL13 contributed positive, albeit weaker, loadings to PC3 [49]. In addition to its large negative projection with respect to PC1, gland P.G5.06.OS also had a large positive projection with respect to PC3, indicating that cells of the PC3-positive loading cluster were present in that gland. In contrast, gland P.G5.01.OS had a negative projection with respect to PC3. Moreover, principal component analysis of transcript abundances in the microdissected immune cell cluster samples only (Figure 8 and Table 2) indicated that mRNAs for CD3ζ, CD80, and CXCL13 contributed strong loadings to the major PC, of the same sign as the loadings contributed by mRNAs for CCL21, CD4, CD25, CD25, CTLA-4, BAFF, and MMP-9.

### 2.4. Cells Outside the Acinus Duct/Immune Cell Cluster Axis

**Figure 9:**
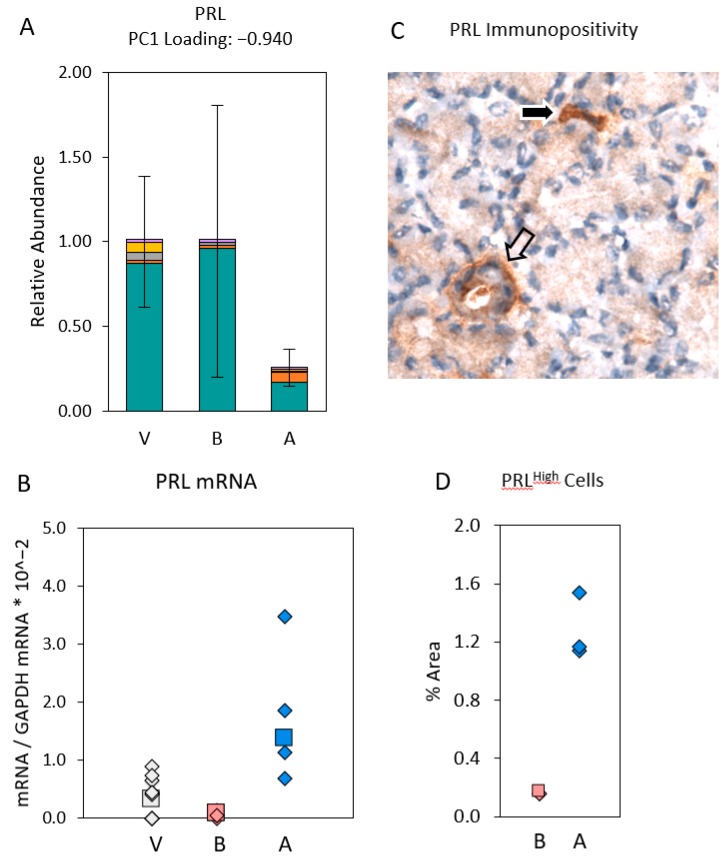
(**A**) Modeled abundances of mRNA for PRL in epithelial segment samples and immune cell samples dissected from the P.G5.B glands, the P.G5.A gland, and a V.G5 gland. Color code and error bars are as defined in Figure 6. (**B**) Measured abundances of mRNA for PRL (PRL mRNA/GAPDH mRNA) in all V.G5, P.G5.B, and P.G5.A glands. Glands selected for microdissection are indicated by large-font symbols. (**C**) Frozen section stained for prolactin (PRL) immunoreactivity. The black arrow indicates an intensely immunopositive cell (PRL^High^ cell) in the interacinar space. The white arrow indicates a duct. (**D**) Percent areas occupied by PRL^High^ cells in three P.G5.B glands and three P.G5.A glands.

The modeled abundances of mRNA for PRL were remarkably disparate from the measured abundances of mRNA for PRL in the whole-gland samples (Figure 9). The modeled abundance of mRNA PRL for gland P.G5.06.OS samples was much lower than the modeled abundances for samples that were microdissected from a V.G5 gland [47]. This finding is consistent with the hypothesis that epithelial cells in gland P.G5.06.OS downregulated their expression of PRL in response to the high levels of PRL that the anterior pituitary contributes to the circulation during pregnancy. The large variances of the modeled abundances in microdissected samples from the P.G5.B gland precludes a comparison. However, the measured abundance of mRNA for PRL in gland P.G5.06.OS sample was much higher than in the P.G5.B whole-gland samples and the V.G5 whole-gland samples. This finding suggests that high levels of PRL were expressed by cells that we did not sample by microdissection of gland P.G5.06.OS. To test this hypothesis, we stained frozen sections of three P.G5.B glands and three P.G5.A glands for PRL immunopositivity. Cells, possibly macrophages [51], that were intensely positive for PRL immunoreactivity were scattered in interacinar spaces, where they would not have been sampled by our protocol, and they were 8.15-fold more frequent (*p* < 0.001) in gland P.G5.06.OSs than in the P.G5.B glands (Figure 9). The finding of a high frequency of PRL^High^ cells in P.G5.A glands is consistent with the hypothesis that cells outside the acinus duct axis participated in the Sjögren’s-like epithelium immune cell network.

## 3. Discussion

Our findings indicate that clusters of immune cells expressing high levels of many of the same transcripts that are highly expressed in Sjögren’s infiltrates are present in histologically normal lacrimal glands. As the clusters can be detected before classical Sjögren’s infiltrates are identified, we suggest that they may be early precursors of Sjögren’s infiltrates. This conjecture is consistent with the finding that levels of the typical Sjögren’s autoantibodies can be elevated before Sjögren’s syndrome symptoms become clinically significant [52]. 

Our findings are consistent with the hypothesis that signaling interactions with epithelial cells in all levels of the acinus duct axis, as well as with cells outside the acinus duct axis, influence formation of the immune cell clusters that evade peripheral tolerance mechanisms. Acinar cells maintain high baseline levels of APRIL. Acinar and ductal cells appear to contribute CCL2, CCL4, CCL28, BAFF, IL-6, and IL-10. Immune cell clusters appear to contribute CXCL13, CCL21, MHC II, and MMP-9 [53], as well as additional BAFF. The levels at which mRNAs for these proteins are expressed vary coordinately, and through a wide range, as indicated by the range of gland projections with respect to PC1 (Figure 2).

Because mRNA for MHC II was predominantly expressed in immune cell clusters, it appears that induction of epithelial HLA class II molecule expression may be a downstream event in Sjögren’s pathogenesis, rather than a triggering event in Sjögren’s etiology. Notably, however, acinar epithelial cells expressed mRNAs for MHC I, CD1d, CD80, and CD86. Therefore, lacrimal gland epithelial cells might contribute to Sjögren’s etiology not only by expressing chemokines that recruit T cells, B cells, and professional antigen-presenting cells, but also by expressing mitogenic factors that support immune cell survival and CD4^+^ cell proliferation in response to MHC II-restricted epitopes presented by the professional antigen-presenting cells. They might also by displaying costimulatory signals, presenting MHC I-bound autoantigen epitopes to CD8^+^ T cells, and presenting CD1d-bound glycolipids to invariant α-chain NK T cells. Presumably, the antigen receptors of CD4^+^ or CD8^+^ T cells that participate in such interactions would have autoantigen epitope affinities strong enough to support positive selection during thymic maturation, but too weak to determine activation-induced deletion. 

A corollary to this hypothesis is that, in addition to the coreceptors, CD80 and CD86, epithelial cell also express additional coreceptors and receptors, typically expressed by T cells, which mediate immune cell-to-epithelial cell paracrine signaling, as well as epithelial cell autocrine signaling and epithelial cell-to-epithelial cell paracrine signaling. As indicated in Figure 5, epithelial cells expressed mRNA for CD25, the α-subunit of the IL-2 receptor; mRNA for the CCL4 receptor, CCR5; mRNAs for CD8 and CD28 (Figure 6); and mRNA for IL-2 [47]. These transcripts all contributed to the PC1-negative loading transcript cluster. These findings are not unprecedented, as CD8 is known to be expressed by NK cells, dendritic cells, and cortical thymocytes, as well as by T cells; CD28 is known to be expressed by plasmacytoid dendritic cells, plasmacytes, NK cells, eosinophils, and neutrophils, as well as by T cells [54]; and IL-2 receptors are known to be expressed by renal tubular epithelial cells [55,56]. Their expression by epithelial cells in the lacrimal gland might contribute to the positive feedback loop which coordinates epithelial cell expression and immune cell expression of the transcripts of the PC1-negative loading cluster.

The resemblance between the transcript expression profiles of immune cell clusters and Sjögren’s foci is likely even greater than revealed by the present microdissection data, as PC1 also receives strong negative loadings from additional transcripts that also are expressed in Sjögren’s foci. The transcripts that contribute strong negative loadings to PC1 include CD40L and CD40 [49], a cognate coreceptor pair expressed respectively by T cells and by B cells and professional antigen-presenting cells. They also include mRNAs for CD19 and CD72 [49], expressed by resting B cells; mRNAs for IL-2, IL-4, IL-5, IL-7, IL-13, IL-17A [49], which contribute to B-cell activation; and mRNA for IL-21 [49], which supports T-cell survival in T-cell zones and dark zone bases. 

Several transcripts that are particularly associated with active lymphoid follicles notably do not contribute strong negative loadings to PC1. These include mRNA for lymphotoxin beta (LT-β), expressed by lymphoid tissue-organizing cells; mRNA for CXCL12, which recruits B cells to germinal center dark zones; mRNA for amyloid precursor protein intracellular domain (AICD), which mediates immunoglobulin gene somatic hyper mutation; mRNA for CD22, expressed by mature B cells; and mRNA for CD138, expressed by terminally differentiated plasmacytes [49]. Each of these transcripts contributes a strong or moderately strong negative loading to PC2. Gland P.G5.06.OS had a positive projection with respect to PC2, but gland P.G5.03.OD, another subgroup P.G5.A gland that had an even larger negative projection with respect to PC1, had a large negative projection with respect to PC2. Our finding (Figure 7) that immune cell cluster samples from gland P.G5.06.OS had significantly higher abundances of transcripts that contributed strong loadings to other PCs is informative in this context, as it indicates that transcripts which contribute to other PCs can be expressed in the same immune cell clusters as the PC1-negative loading transcripts. In other words, immune cell clusters in gland P.G5.03.OD may have been relatively further along on a trajectory toward manifestation as recognizable ectopic lymphoid structures with germinal centers. Moreover, because autoreactive B cells escape self-recognition checkpoints when they are activated in ectopic lymphoid structures [57,58,59], one might ask whether B cells in the immune cell clusters we described contribute autoantibodies to the circulation even before germinal centers are formed.

The remarkably broad distribution of lacrimal glands’ projections with respect to PC1 as depicted in Figure 2 may offer a hint for answering the question of why the prevalence of primary Sjögren’s syndrome is relatively low (0.02–0.1%) [60] That is, immune cell clusters are only likely to expand into Sjögren’s foci in the glands in which they are already well developed, i.e., in glands with the largest negative projections with respect to PC1. In view of the broad distributions of lacrimal glands’ projections with respect to PC2 and PC3, and the broad distributions of subgroup P.G5.A glands’ projections with respect to PC4 and PC5 as depicted in Figure 2, the conclusion that immune cell clusters express transcripts from multiple transcript clusters may have general implications for understanding the heterogeneities of both primary and secondary Sjögren’s syndrome phenotypes. 

In rabbits, the likelihood that a gland will achieve a large negative PC1 projection is increased by exposure to high degrees of environmental dryness, as well as by the hormonal environment of pregnancy (Figure 3). Environmental dryness is a risk factor for dry-eye disease in humans [61]; to our knowledge, it is yet to be reported as a risk factor for Sjögren’s syndrome. However, the findings in Figure 2, Figure 3 and Figure 4 demonstrate that responses to both environmental dryness and the hormonal environment of pregnancy are subject to very high levels of stochasticity.

In addition to the genetic risk factors we discussed in Section 1 of this paper, mechanisms related to viral infections were proposed [62,63,64,65,66]; these include mimicry of autoantigens by viral proteins, apoptosis-associated release of autoantigens, apoptosis-associated release of ligands for molecular pattern recognition receptors, and expression of proliferation factors and chemokines in latently infected T cells or B cells [67,68]. Occupational exposure to organic solvents [69] and exposure to psychosocial stress [70] were also proposed to be risk factors. Our findings indicate that epithelial cells in histologically normal lacrimal glands are able to recruit immune cells, provide mitogenic support for them, and shape their activities. These abilities may contribute to the responses to such risk factors, and the inherent stochasticity of the development of immune cell/epithelial cell networks would contribute to the stochasticity of responses to the risk factors.

## 4. Methods

### 4.1. Animals

We analyzed lacrimal glands from six groups of female rabbits, all described previously [47,48,49,50]. Groups V.G2, V.G3, V.G5, and V.G6 were 20-week-old nulliparous adults. Incomplete data were obtained from a sixth group of 20-week-old rabbits (V.G4) and were not included in the analysis. Group P.G5 was term-pregnant 20-week-old rabbits. Group V.G7 was 52-week-old nulliparous adults [50]. Groups V.G2, V.G3, V.G5, P.G5, and V.G6 were obtained from a barrier-free facility (Irish Farms, Norco, CA, USA). They were raised at different times and, thus, were exposed to different environmental temperatures and different degrees of environmental dryness prior to arriving at the University of Southern California Health Sciences Campus Vivaria (USC HSC, Los Angeles, CA, USA) for a four-day acclimation period. Group V.G7 was raised and maintained in barrier facilities (Covance Research Products, Denver, PA, and the USC HSC Vivaria), where the animals were exposed to consistently mild temperatures and mild degrees of dryness. All procedures with animals conformed with the Association for Research in Vision and Ophthalmology (ARVO) statement on the Use of Animals in Vision Research. All procedures with animals were approved by the University of Southern California Institutional Animal Care and Use Committee, protocol #20057, 24 August 2016.

### 4.2. Tissue Collection

We euthanized rabbits with Euthasol^®^ after sedating with ketamine/xylazine. We removed lacrimal glands at necropsy in RNAse-free conditions and divided each gland into three parts. We placed one part in RNALater^®^ for RNA extraction, one part in formalin for paraffin embedding and hematoxylin and eosin (H&E) and trichrome staining studies (not reported), and one part in OCT^®^ for rapid freezing in liquid nitrogen, sectioning, immunohistochemical staining, and laser capture microdissection. 

### 4.3. Laser Capture Microdissection

We collected frozen sections on membrane-coated slides (PEN^®^; Leica Microsystems, Deerfield, IL, USA) and stained them with cresyl violet from Applied Biosystems (Foster City, CA, USA) [71]. We then microdissected samples of epithelial cells from acini; samples of epithelial cells from intralobular ducts, interlobular ducts, and intralobar ducts; and samples of immune cells from clusters around interlobular ducts using the PixCell II LCM System^®^ (Arcturus Bioscience, Mountain View, CA, USA) and Cap-Sure HS^®^ laser capture microdissection caps from Molecular Devices, Sunnyvale, CA, USA. We collected five or six replicate samples of the epithelial segments and five samples of immune cell clusters from each gland; each sample contained approximately 100 cells. 

### 4.4. Real-Time Reverse-Transcriptase Polymerase Chain Reaction (Real Time RT-PCR)

We extracted mRNA with RNAqueous Micro^®^ kits from Ambion (Austin, TX, USA) according to the manufacturer’s protocol, eluting with 11-L aliquots of prewarmed elution solutions. After treatment with DNAase and quality control with an ND-1000 spectrophotometer from Nanodrop Technologies (Wilmington, DE, USA), we reversed-transcribed to complementary DNA (cDNA) with High-Capacity cDNA Reverse Transcription Kits and RNase Inhibitor from Applied Biosystems using a DNA Engine^®^ thermal cycler from Bio-Rad (Hercules, CA, USA). We preamplified cDNA using TaqMan PreAmp Master Mix^®^ from Applied Biosystems. We performed real-time RT-PCR with a Prism 790HT^®^ from Applied Biosystems using primer and probe sequences selected with the Primer Express^®^ program and synthesized by Applied Biosystems. We expressed all transcript abundances relative to the abundance of mRNA for glyceraldehyde 3-phosphate dehydrogenase (GAPDH).

### 4.5. Immunohistochemical Staining

We used the protocol described by Ding et al. to stain frozen sections for PRL immunopositivity [46].

### 4.6. Data Analysis

As in previous studies [49,50], we submitted relative transcript abundances to Partek Genomics Suite^®^ for principal component analysis, and we accepted all principal components with eigenvalues >1.0 as significant. We used SigmaPlot 14 for nonlinear regression analyses, ANOVA for differences between means of normally distributed values, and the Kruskal–Wallis one-way analysis of variance on ranks for differences between non-normally distributed values.

## 5. Conclusions

If immune cell cluster/epithelial cell networks analogous to the networks we described develop in human lacrimal glands, the ability to detect their molecular signatures would have important implications for the diagnosis of incipient Sjögren’s foci and for the design and implementation of therapies to prevent progression to clinically significant pathology.

## Figures and Tables

**Table 1:** Transcript Loadings to Significant Principal Components.

Variable	PC 1	Variable	PC 2	Variable	PC 3	Variable	PC 4	Variable	PC 5
CCL28	0.2729	CCL21	−0.9465	CCL2	−0.8706	TNF-a	−0.7861	CCL4	−0.2317
MMP-9	0.0677	IL-18	−0.9337	MHC II	−0.8296	CD4	−0.5162	IL-6	−0.1182
IL-18	0.0516	MMP-9	−0.9093	CCL4	−0.5254	CD8	−0.2194	IL-10	−0.1129
CCL21	0.0250	PRL	−0.7501	TNF-a	−0.3396	CD28	−0.2127	BAFF	−0.1065
TNF-a	−0.0210	BAFF	−0.4072	CTLA-4	−0.2869	IL-18	−0.1159	PRL	−0.0890
CXCL13	−0.3782	MHC II	−0.1641	IL-1a	−0.2599	CCL21	−0.1053	CCR5	−0.0661
CCL2	−0.3901	CCL2	−0.1145	MMP-9	−0.0866	CTLA-4	−0.0938	CD4	−0.0614
MHC II	−0.4494	CD4	−0.0912	CXCL13	−0.0763	CXCL13	−0.0133	IL-1a	−0.0458
PRL	−0.5794	CD28	−0.0561	CCL21	−0.0381	MMP-9	0.0682	CCL21	−0.0397
BAFF	−0.6719	CTLA-4	−0.0027	PRL	0.1012	BAFF	0.0720	CCL2	0.0190
CD4	−0.7205	CXCL13	0.0184	IL-1b	0.1161	MHC II	0.0929	MHC II	0.0314
CCL4	−0.7319	IL-10	0.0496	CCR5	0.1804	IL-6	0.1006	IL-18	0.0832
CTLA-4	−0.8066	CCL4	0.0552	IL-18	0.1815	PRL	0.1062	CD8	0.0882
CD8	−0.8348	CCL28	0.0898	CCL28	0.1888	IL-1a	0.1293	CTLA-4	0.1373
CD28	−0.8400	IL-1a	0.0956	IL-6	0.2037	CCL4	0.1308	CD28	0.1912
IL-10	−0.8485	CCR5	0.1113	CD4	0.2227	CCL2	0.1448	MMP-9	0.2257
IL-1b	−0.8651	IL-6	0.1369	CD8	0.2370	CCR5	0.1518	TNF-a	0.2653
CCR5	−0.9154	TNF-α	0.1684	CD28	0.2759	IL-1b	0.1657	IL-1b	0.2748
IL-6	−0.9262	IL-1b	0.2006	BAFF	0.3343	IL-10	0.1798	CXCL13	0.6024
IL-1a	−0.9291	CD8	0.2979	IL-10	0.3915	CCL28	0.4592	CCL28	0.6365

**Table 2:** Transcript Loadings to Immune Cell Cluster Principal Components.

Variable	PC 1	Variable	PC 2	Variable	PC 3	Variable	PC 4	Variable	PC 5	Variable	PC 6
EGF	−0.9669	CD86	−0.8548	MHC II	−0.8895	Lipoph CL	−0.7080	IGF-1B	−0.7061	IL-10	−0.5272
MHC I	−0.9306	CD1d	−0.8354	IL-1a	−0.6157	IL-6	−0.5981	TGF-b2	−0.5759	CCL28	−0.5047
APRIL	−0.9263	CD8	−0.7284	PRL	−0.5481	IL-10	−0.5372	CD80	−0.5058	Decorin	−0.3251
TGF-b2	−0.7619	Decorin	−0.6752	Decorin	−0.4931	IGF-1B	−0.3973	IL-18	−0.5025	CCL2	−0.3127
CCL4	−0.6012	Lipoph CL	−0.4969	CD25	−0.4266	CD28	−0.3191	TGF-b1	−0.3276	IL-6	−0.2676
IL-6	−0.5577	CCL4	−0.4626	CCL28	−0.4064	Decorin	−0.2763	CD28	−0.1887	BAFF	−0.2245
IGF-1B	−0.4630	CCL28	−0.4518	Lipoph CL	−0.3398	CCR5	−0.2163	Decorin	−0.1561	IL-18	−0.1994
CCL2	−0.2098	BAFF	−0.4488	IL-6	−0.2717	APRIL	−0.1796	MMP9	−0.1298	CCL4	−0.1507
CD86	−0.1466	IGF-1B	−0.3372	CTLA-4	−0.2486	IL-1RA	−0.1577	MHC I	−0.1260	CD3z	−0.1246
IL-10	−0.0836	CCL2	−0.3210	APRIL	−0.2257	MHC II	−0.1448	EGF	−0.1161	CD80	−0.1122
Decorin	−0.0128	CCR5	−0.2810	EGF	−0.1710	CCL21	−0.1441	CD25	−0.1106	MHC II	−0.0921
CD8	−0.0095	MHC I	−0.2544	CD86	−0.1617	CD4	−0.1188	APRIL	−0.0994	CTLA-4	−0.0863
CCR5	0.0799	PRL	−0.2342	IL-18	−0.1556	CD86	−0.1145	BAFF	−0.0947	MMP9	−0.0648
MHC II	0.1667	CD80	−0.1728	CD1d	−0.1345	CD8	−0.1125	Lipoph CL	−0.0768	CD25	−0.0532
Lipoph CL	0.2042	pIgR	−0.1648	MHC I	−0.1339	TGF-b1	−0.1108	CCL28	−0.0418	CCR5	−0.0466
IL-1a	0.2462	TGF-b1	−0.1478	CXCL13	−0.1124	CD3z	−0.0994	CTLA-4	−0.0407	APRIL	−0.0448
CCL28	0.3279	MMP9	−0.1086	TGF-b2	−0.1007	EGF	−0.0816	CD3e	−0.0385	CD1d	−0.0284
CD1d	0.4557	APRIL	−0.0702	pIgR	−0.0436	MMP9	−0.0549	CCL4	−0.0275	CD28	−0.0282
PRL	0.5574	CCL21	−0.0211	CD3z	−0.0419	pIgR	−0.0253	CD3z	−0.0120	IL-1RA	−0.0239
CD28	0.7298	EGF	−0.0137	CD3e	0.0378	BAFF	−0.0240	CXCL13	−0.0042	CD4	0.0144
CD25	0.7883	CD3z	0.0071	IL-10	0.0487	TGF-b2	−0.0121	pIgR	−0.0002	TGF-b2	0.0481
IL-18	0.7890	IL-10	0.0082	MMP9	0.0510	CXCL13	−0.0049	IL-1a	0.0355	TGF-b1	0.0605
BAFF	0.7992	CXCL13	0.0388	IL-1RA	0.0624	CD80	−0.0029	CD4	0.0361	CCL21	0.0724
CD80	0.8063	CD25	0.0636	IGF-1B	0.0842	MHC I	0.0216	CD1d	0.0551	IGF-1B	0.0754
TGF-β1	0.9121	MHC II	0.0790	CCL21	0.0901	IL-18	0.0328	CCL2	0.0847	CXCL13	0.0889
IL-1RA	0.9199	CD3e	0.1110	TGF-b1	0.0920	CD3e	0.0533	IL-6	0.0866	MHC I	0.0939
pIgR	0.9225	TGF-b2	0.1111	CCL4	0.1049	CTLA-4	0.1141	CD8	0.1309	EGF	0.0973
CTLA-4	0.9261	CD4	0.1112	CD4	0.1178	CD25	0.1266	CD86	0.1542	CD3e	0.1127
CCL21	0.9589	CD28	0.1157	BAFF	0.1920	CD1d	0.1957	CCL21	0.1991	CD86	0.1171
CXCL13	0.9673	CTLA-4	0.1425	CD80	0.2099	PRL	0.2016	IL-1RA	0.2002	Lipoph CL	0.1818
CD3e	0.9734	IL-18	0.2005	CD8	0.3040	IL-1a	0.4265	PRL	0.2299	pIgR	0.1999
CD3z	0.9749	IL-1RA	0.2385	CCR5	0.3410	CCL28	0.4501	MHC II	0.2489	IL-1a	0.3058
CD4	0.9761	IL-6	0.3726	CCL2	0.3749	CCL4	0.4738	IL-10	0.4431	PRL	0.4001
MMP9	0.9795	IL-1a	0.4557	CD28	0.5472	CCL2	0.7207	CCR5	0.7383	CD8	0.5382

## References

[1] Moutsopoulos H.M., Webber B.L., Vlagopoulos T.P., Chused T.M., Decker J.L. (1979). Differences in the clinical manifestations of sicca syndrome in the presence and absence of rheumatoid arthritis. Am. J. Med..

[2] Christodoulou M.I., Kapsogeorgou E.K., Moutsopoulos H.M. (2010). Characteristics of the minor salivary gland infiltrates in Sjögren’s syndrome. J. Autoimmun..

[3] Maria N.I., Vogelsang P., Versnel M.A. (2015). The clinical relevance of animal models in Sjögren’s syndrome: The interferon signature from mouse to man. Arthritis Res. Ther..

[4] Kapsogeorgou E.K., Christodoulou M.I., Panagiotakos D.B., Paikos S., Tassidou A., Tzioufas A.G., Moutsopoulos H.M. (2013). Minor salivary gland inflammatory lesions in Sjögren syndrome: Do they evolve?. J. Rheumatol..

[5] Chused T.M., Kassan S.S., Opelz G., Moutsopoulos H.M., Terasaki P.I. (1977). Sjögren’s syndrome association with HLA-Dw3. N. Engl. J. Med..

[6] Moutsopoulos H.M., Chused T.M., Johnson A.H., Khudsen B., Mann D.L. (1978). B lymphocyte antigens in sicca syndrome. Science.

[7] Gottenberg J.E., Busson M., Loiseau P., Cohen-Solal J., Lepage V., Charron D., Sibilia J., Mariette X. (2003). In primary Sjögren’s syndrome, HLA class II is associated exclusively with autoantibody production and spreading of the autoimmune response. Arthritis Rheum..

[8] Font J., Garcia-Carrasco M., Ramos-Casals M., Aldea A.I., Cervera R., Ingelmo M., Vives J., Yague J. (2002). The role of interleukin-10 promoter polymorphisms in the clinical expression of primary Sjögren’s syndrome. Rheumatology.

[9] Anaya J.M., Correa P.A., Herrera M., Eskdale J., Gallagher G. (2002). Interleukin 10 (IL-10) influences autoimmune response in primary Sjogren’s syndrome and is linked to IL-10 gene polymorphism. J. Rheumatol..

[10] Hulkkonen J., Pertovaara M., Antonen J., Lahdenpohja N., Pasternack A., Hurme M. (2001). Genetic association between interleukin-10 promoter region polymorphisms and primary Sjögren’s syndrome. Arthritis Rheum..

[11] Gottenberg J.E., Busson M., Loiseau P., Dourche M., Cohen-Solal J., Lepage V., Charron D., Miceli C., Sibilia J., Mariette X. (2004). Association of transforming growth factor beta1 and tumor necrosis factor alpha polymorphisms with anti-SSB/La antibody secretion in patients with primary Sjogren’s syndrome. Arthritis Rheum..

[12] Kumagai S., Kanagawa S., Morinobu A., Takada M., Nakamura K., Sugai S., Maruya E., Saji H. (1997). Association of a new allele of the TAP2 gene, TAP2*Bky2 (Val577), with susceptibility to Sjogren’s syndrome. Arthritis Rheum..

[13] Li H., Reksten T.R., Ice J.A., Kelly J.A., Adrianto I., Rasmussen A., Wang S., He B., Grundahl K.M., Glenn S.B. (2017). Identification of a Sjögren’s syndrome susceptibility locus at OAS1 that influences isoform switching, protein expression, and responsiveness to type I. interferons. PLoS Genet..

[14] Helmick C.G., Felson D.T., Lawrence R.C., Gabriel S., Hirsch R., Kwoh C.K., Liang M.H., Kremers H.M., Mayes M.D., Merkel P.A. (2008). National Arthritis Data Workgroup. Estimates of the prevalence of arthritis and other rheumatic conditions in the United States. Part, I. Arthritis Rheum..

[15] Priori R., Medda E., Conti F., Cassarà E.A., Sabbadini M.G., Antonioli C.M., Gerli R., Danieli M.G., Giacomelli R., Pietrogrande M. (2007). Risk factors for Sjögren’s syndrome: A case-control study. Clin. Exp. Rheumatol..

[16] Jørgensen K.T., Pedersen B.V., Nielsen N.M., Jacobsen S., Frisch M. (2012). Childbirths and risk of female predominant and other autoimmune diseases in a population based Danish cohort. J. Autoimmun..

[17] Anaya J.M., Delgado-Vega A.M., Castiblanco J. (2006). Genetic basis of Sjögren’s syndrome. How strong is the evidence?. Clin. Dev. Immunol..

[18] Hanafusa T., Pujol-Borrell R., Chiovato L., Russell R.C.G., Doniach D., Bottazzo G.F. (1983). Aberrant expression of HLA-DR antigen on thyrocytes in Graves’ disease: Relevance for autoimmunity. Lancet.

[19] Bottazzo G.F., Pujol-Borrell R., Hanafusa T. (1983). Role of aberrant HLA-DR expression and antigen presentation in induction of endocrine autoimmunity. Lancet.

[20] Lindahl G., Hedfors E., Klareskog L., Forsum U. (1985). Epithelial HLA-DR expression and T lymphocyte subsets in salivary glands in Sjögren’s syndrome. Clin. Exp. Immunol..

[21] Fox R.I., Bumol T., Fantozzi R., Bone R., Schreiber R. (1986). Expression of histocompatibility antigen HLA-DR by salivary gland epithelial cells in Sjogren’s syndrome. Arthritis Rheum..

[22] Moutsopoulos H.M., Hooks J.J., Chan C.C., Dalavanga Y.A., Skopouli F.N., Detrick B. (1986). HLA-DR expression by labial minor salivary gland tissues in Sjögren’s syndrome. Ann. Rheum. Dis..

[23] Rowe D., Griffiths M., Stewart J., Novick D., Beverley P.C., Isenberg D.A. (1987). HLA class I and II, interferon, interleukin 2, and the interleukin 2 receptor expression on labial biopsy specimens from patients with Sjögren’s syndrome. Ann. Rheum. Dis..

[24] Manoussakis M.N., Dimitriou I.D., Kapsogeorgou E.K., Xanthou G., Paikos S., Polihronis M., Moutsopoulos H.M. (1999). Expression of B7 costimulatory molecules by salivary gland epithelial cells in patients with Sjögren’s syndrome. Arthritis Rheum..

[25] Dimitriou I.D., Kapsogeorgou E.K., Moutsopoulos H.M., Manoussakis M.N. (2002). CD40 on salivary gland epithelial cells: High constitutive expression by cultured cells from Sjögren’s syndrome patients indicating their intrinsic activation. Clin. Exp. Immunol..

[26] Cuello C., Palladinetti P., Tedla N., Di Girolamo N., Lloyd A.R., McCluskey P.J., Wakefield D. (1998). Chemokine expression and leucocyte infiltration in Sjögren’s syndrome. Br. J. Rheumatol..

[27] Xanthou G., Polihronis M., Tzioufas A.G., Paikos S., Sideras P., Moutsopoulos H.M. (2001). “Lymphoid” chemokine messenger RNA expression by epithelial cells in the chronic inflammatory lesion of the salivary glands of Sjögren’s syndrome patients: Possible participation in lymphoid structure formation. Arthritis Rheum..

[28] Ruffilli I. (2014). Sjögren’s syndrome and chemokines. Clin. Ther..

[29] Ogawa N., Ping L., Zhenjun L., Takada Y., Sugai S. (2002). Involvement of the interferon-gamma-induced T cell-attracting chemokines, interferon-gamma-inducible 10-kd protein (CXCL10) and monokine induced by interferon-gamma (CXCL9), in the salivary gland lesions of patients with Sjögren’s syndrome. Arthritis Rheum..

[30] Latchney L.R., Fallon M.A., Culp D.J., Gelbard H.A., Dewhurst S. (2004). Immunohistochemical assessment of fractalkine, inflammatory cells, and human herpesvirus 7 in human salivary glands. J. Histochem. Cytochem..

[31] Lee J.H., Kwok S.K., Jung S.M., Lee J., Lee J.S., Baek S.Y., Kim E.K., Ju J.H., Park S.H., Kim H.Y. (2014). Role of fractalkine in the pathogenesis of primary Sjögren syndrome: Increased serum levels of fractalkine, its expression in labial salivary glands, and the association with clinical manifestations. J. Rheumatol..

[32] Barone F., Bombardieri M., Rosado M.M., Morgan P.R., Challacombe S.J., De Vita S., Carsetti R., Spencer J., Valesini G., Pitzalis C. (2008). CXCL13, CCL21, and CXCL12 expression in salivary glands of patients with Sjogren’s syndrome and MALT lymphoma: Association with reactive and malignant areas of lymphoid organization. J. Immunol..

[33] Berri M., Meurens F., Lefevre F., Chevaleyre C., Zanello G., Gerdts V., Salmon H. (2008). Molecular cloning and functional characterization of porcine CCL28: Possible involvement in homing of IgA antibody secreting cells into the mammary gland. Mol. Immunol..

[34] Cauli A., Yanni G., Pitzalis C., Challacombe S., Panayi G.S. (1995). Cytokine and adhesion molecule expression in the minor salivary glands of patients with Sjögren’s syndrome and chronic sialoadenitis. Ann. Rheum. Dis..

[35] Oxholm P., Daniels T.E., Bendtzen K. (1992). Cytokine expression in labial salivary glands from patients with primary Sjögren’s syndrome. Autoimmunity.

[36] Bombardieri M., Barone F., Pittoni V., Alessandri C., Conigliaro P., Blades M.C., Priori R., McInnes I.B., Valesini G., Pitzalis C. (2004). Increased circulating levels and salivary gland expression of interleukin-18 in patients with Sjögren’s syndrome: Relationship with autoantibody production and lymphoid organization of the periductal inflammatory infiltrate. Arthritis Res. Ther..

[37] Daridon C., Devauchelle V., Hutin P., Le Berre R., Martins-Carvalho C., Bendaoud B., Dueymes M., Saraux A., Youinou P., Pers J.O. (2007). Aberrant expression of BAFF by B lymphocytes infiltrating the salivary glands of patients with primary Sjögren’s syndrome. Arthritis Rheum..

[38] Sun D., Emmert-Buck M.R., Fox P.C. (1998). Differential cytokine mRNA expression in human labial minor salivary glands in primary Sjögren’s syndrome. Autoimmunity.

[39] Ha Y.J., Choi Y.S., Kang E.H., Chung J.H., Cha S., Song Y.W., Lee Y.J. (2018). Increased expression of interferon-λ in minor salivary glands of patients with primary Sjögren’s syndrome and its synergic effect with interferon-α on salivary gland epithelial cells. Clin. Exp. Rheumatol..

[40] Frey W.H., Nelson J.D., Frick M.L., Eide R.P., Holly F.J. (1986). Prolactin immunoreactivity in human tears and lacrimal gland: Possible implications for tear production. The Preocular Tear Film in Health, Disease, and Contact Lens Wear.

[41] Hartmann D.P., Holaday J.W., Bernton E.W. (1989). Inhibition of lymphocyte proliferation by antibodies to prolactin. FASEB J..

[42] Montgomery D.W., LeFevre J.A., Ulrich E.D., Adamson C.R., Zukowski C.F. (1990). Identification of prolactin-like proteins synthesized by normal murine lymphocytes. Endocrinology.

[43] Peeva E., Venkatesh J., Michael D., Diamond B. (2004). Prolactin as a modulator of B cell function: Implications for SLE. Biomed. Pharmacother..

[44] Jara L.J., Medina G., Saavedra M.A., Vera-Lastra O., Torres-Aguilar H., Navarro C., Vazquez Del Mercado M., Espinoza L.R. (2017). Prolactin has a pathogenic role in systemic lupus erythematosus. Immunol. Res..

[45] Schechter J., Carey J., Wallace M., Wood R. (2000). Distribution of growth factors and immune cells are altered in the lacrimal gland during pregnancy and lactation. Exp. Eye Res..

[46] Ding C., Chang N., Fong Y.C., Wang Y., Trousdale M.D., Mircheff A.K., Schechter J.E. (2006). Interacting influences of pregnancy and corneal injury on rabbit lacrimal gland immunoarchitecture and function. Investig. Ophthalmol. Vis. Sci..

[47] Mircheff A.K., Wang Y., Ding C., Warren D.W., Schechter J.E. (2015). Potentially pathogenic immune cells and networks in apparently healthy lacrimal glands. Ocul. Surf..

[48] Mircheff A.K., Wang Y., Thomas P.B., Nakamura T., Samant D., Trousdale M.D., Warren D.W., Ding C., Schechter J.E. (2011). Systematic variations in immune response-related gene transcript abundance suggest new questions about environmental influences on lacrimal gland immunoregulation. Curr. Eye Res..

[49] Mircheff A.K., Wang Y., Li M., Pan B.X., Ding C. (2018). Pregnancy probabilistically augments potential precursors to chronic, immune-mediated or autoimmune lacrimal gland infiltrates. Ocul. Surf..

[50] Mircheff A.K., Wang Y., Schechter J.E., Li M., Tong W., Attar M., Chengalvala M., Harmuth J., Prusakiewicz J.J. (2016). Multiple natural and experimental inflammatory rabbit lacrimal gland phenotypes. Ocul. Surf..

[51] Tang M.W., Garcia S., Malvar Fernandez B., Gerlag D.M., Tak P.P., Reedquist K.A. (2017). Rheumatoid arthritis and psoriatic arthritis synovial fluids stimulate prolactin production by macrophages. J. Leukoc. Biol..

[52] Jonsson R., Theander E., Sjöström B., Brokstad K., Henriksson G. (2013). Autoantibodies present before symptom onset in primary Sjögren syndrome. JAMA.

[53] Konttinen Y., Halinen S., Hanemaaijer R., Sorsa T., Hietanen J., Ceponis A., Xu J.W., Manthorpe R., Whittington J., Larsson Å. (1998). Matrix metalloproteinase (MMP)-9 type IV collagenase/gelatinase implicated in the pathogenesis of Sjögren’s syndrome. Matrix Biol..

[54] Macal M., Tam M.A., Hesser C., Di Domizio J., Leger P., Gilliet M., Zuniga E.I. (2016). CD28 deficiency enhances type I IFN production by murine plasmacytoid dendritic cells. J. Immunol..

[55] Wang S., Zhang Z.X., Yin Z., Liu W., Garcia B., Huang X., Acott P., Jevnikar A.M. (2011). Anti-IL-2 receptor antibody decreases cytokine-induced apoptosis of human renal tubular epithelial cells (TEC). Nephrol. Dial. Transplant..

[56] Tejman-Yarden N., Zlotnik M., Lewis E., Etzion O., Chaimovitz C., Douvdevani A. (2005). Renal cells express a functional interleukin-15 receptor. Nephrol. Dial. Transplant..

[57] Lanzavecchia A., Sallusto F. (2007). Toll-like receptors and innate immunity in B-cell activation and antibody responses. Curr. Opin. Immunol..

[58] Guerrier T., Le Pottier L., Devauchelle V., Pers J.O., Jamin C., Youinou P. (2012). Role of Toll-like receptors in primary Sjögren’s syndrome with a special emphasis on B-cell maturation within exocrine tissues. J. Autoimmun..

[59] Nocturne G., Mariette X. (2018). B cells in the pathogenesis of primary Sjögren syndrome. Nat. Rev. Rheumatol..

[60] Maciel G., Crowson C.S., Matteson E.L., Cornec D. (2017). Prevalence of primary Sjögren’s syndrome in a US population-based cohort. Arthritis Care Res..

[61] Paschides C.A., Stefaniotou M., Papageorgiou J., Skourtis P., Psilas K. (1998). Ocular surface and environmental changes. Acta Ophthalmol. Scand..

[62] Fox R.I., Chilton T., Scott S., Benton L., Howell F.V., Vaughan J.H. (1987). Potential role of Epstein–Barr virus in Sjögren’s syndrome. Rheum. Dis. Clin. North Am..

[63] Triantafyllopoulou A., Tapinos N., Moutsopoulos H.M. (2004). Evidence for coxsakie virus infection in primary Sjögren’s syndrome. Arthritis Rheum..

[64] Wang Y., Dou H., Liu G., Yu L., Chen S., Min Y., Zhao K., Wang X., Hu C. (2014). Hepatitis C virus infection and the risk of Sjögren or sicca syndrome: A meta-analysis. Microbiol. Immunol..

[65] Weller M.L., Gardener M.R., Bogus Z.C., Smith M.A., Astorri E., Michael D.G., Michael D.A., Zheng C., Burbelo P.D., Lai Z. (2016). Hepatitis Delta virus detected in salivary glands of Sjögren’s syndrome patients and recapitulates a Sjögren’s syndrome-like phenotype in vivo. Pathog. Immun..

[66] Terada K., Katamine S., Eguchi K., Moriuchi R., Kita M., Shimada H., Yamashita I., Iwata K., Tsuji Y., Nagataki S. (1994). Prevalence of serum and salivary antibodies to HTLV-1 in Sjogren’s syndrome. Lancet.

[67] Ittah M., Miceli-Richard C., Lebon P., Pallier C., Lepajolec C., Mariette X. (2011). Induction of B cell-activating factor by viral infection is a general phenomenon, but the types of viruses and mechanisms depend on cell type. J. Innate Immun..

[68] Croia C., Astorri E., Murray-Brown W., Willis A., Brokstad K.A., Sutcliffe N., Piper K., Jonsson R., Tappuni A.R., Pitzalis C. (2014). Implication of Epstein-Barr virus infection in disease-specific autoreactive B cell activation in ectopic lymphoid structures of Sjögren’s syndrome. Arthritis Rheumatol..

[69] Chaigne B., Lasfargues G., Marie I., Hüttenberger B., Lavigne C., Marchand-Adam S., Maillot F., Diot E. (2015). Primary Sjögren’s syndrome and occupational risk factors: A. case-control study. J. Autoimmun..

[70] Skopouli F.N., Katsiougiannis S. (2018). How stress contributes to autoimmunity-lessons from Sjögren’s syndrome. FEBS Lett..

[71] Ding C., Nandoskar P., Lu M., Thomas P., Trousdale M.D., Wang Y. (2011). Changes of aquaporins in the lacrimal glands of a rabbit model of Sjögren’s syndrome. Curr. Eye Res..

